# Trends in Medicare Reimbursement for Common Pediatric Orthopedic Procedures: From 2000 to 2022

**DOI:** 10.7759/cureus.60062

**Published:** 2024-05-10

**Authors:** Taylor F Faust, Pablo Castaneda

**Affiliations:** 1 Department of Research, Alabama College of Osteopathic Medicine, Dothan, USA; 2 Department of Orthopedic Surgery, Baylor College of Medicine, Houston, USA

**Keywords:** economics of healthcare, physician reimbursement, medicare reimbursement, orthopedic surgery, pediatrics

## Abstract

Objective

We aimed to evaluate trends in government monetary reimbursement (Medicare) for 10 of the most commonly performed pediatric orthopedic procedures from 2000 to 2020.

Methods

Utilizing the Centers for Medicare and Medicaid Services website, we collected data for 10 of the most commonly performed pediatric orthopedic surgical procedures and their variations. The reimbursement data for each procedure was taken from the Current Procedural Terminology (CPT) code, which was collected from the Physician Fee Schedule Look-Up Tool from the Centers for Medicare and Medicaid Services (Baltimore County, MD). The reimbursement values were adjusted for inflation to the 2022 US dollar (USD) using the changes to the Consumer Price Index. The compound annual growth rates (CAGRs) and total percentage changes in reimbursement were calculated for all the procedures and put into relative value units.

Results

Reimbursement for 20 of the 22 total procedures decreased by 32.65% from 2000 to 2022 after adjusting for inflation. Achilles tenotomy with local anesthesia saw the greatest decrease (-54.38%), whereas the procedure revision of spinal fusion saw the highest increase (26.00%) in mean adjusted reimbursement during this study period. Adjusted reimbursement decreased by an average of 2.08% on a yearly basis.

Conclusion

This study is the first to take an in-depth view and evaluate trends in procedural Medicare reimbursement for pediatric orthopedic surgery. When adjusted for inflation, Medicare reimbursement for 20 of 22 included procedures has steadily decreased from 2000 to 2022. There needs to be an increased awareness and consideration of these trends as they will be important for policymakers, hospitals, and surgeons to ensure continued access to meaningful surgical pediatric orthopedic care in the United States.

## Introduction

In the United States, the billing for healthcare is a multifaceted, multipayer system, featuring Medicare as the largest payer [1]. The primary physician billing system is done through the Current Procedural Terminology (CPT), where each code is given a reimbursement value. This can be based on many factors, such as work, the expense of the practice, and malpractice expenses. Through this system, these factors are then quantified into a value, which is then converted into a specific payment rate for each of the CPT codes [2]. In the literature, it has been shown that physicians' earnings have decreased compared to other health professionals [3] and face inflation rate issues [4]. Changing costs of healthcare [5], reimbursement models [6,7], and additional economic uncertainty will alter the constantly evolving healthcare system.

To comprehensively explore the reimbursement landscape for pediatric orthopedic surgeons, this report aims to delve deeper into the reimbursement of pediatric orthopedic procedures as it holds great importance in providing financial sustainability to orthopedic practices. A previous paper, Trends in Medicare Reimbursement for Orthopedic Procedures: 2000 to 2016 [7], examined the adult population's physician fees for orthopedic procedures. In that study, they found that for all procedures in adult orthopedic subspecialties, procedural reimbursement decreased slowly, and the reimbursement rate for procedures in adult reconstruction had the greatest decrease; however, they did not explicitly analyze pediatric orthopedic surgery [8]. Further investigation is needed to demonstrate more insight into physician compensation and healthcare costs. This study aims to evaluate the trends in Medicare reimbursement in pediatric orthopedic surgery between 2000 and 2022.

## Materials and methods

We selected 22 of the most common pediatric orthopedic procedures [6] performed in the United States for evaluation. The CPT codes for several common orthopedic procedures and their associated variations are seen in Table 1.

**Table 1:** The top pediatric orthopedic procedures and variations with the associated Current Procedural Terminology (CPT) codes SCH, supracondylar humerus; ORIF, open reduction and internal fixation

Procedure description	CPT code
Fracture fixation (percutaneous fixation of SCH fracture)	24538
Fracture fixation (ORIF of both-bone fracture)	25575
Posterior spinal fusion (anterior approach for lumbar fusion)	22558
Lower extremity tendon lengthening surgery (osteoplasty, the tibia and fibula, lengthening or shortening)	27715
Revision of spinal fusion (revision including the replacement of total disc arthroplasty)	22861
Achilles tenotomy (local anesthesia)	27605
Achilles tenotomy (general anesthesia)	27606
Arrest, epiphyseal, any method (proximal tibia/fibula)	27477
Treatment of slipped femoral epiphysis (by traction, without reduction, by single or multiple pinning, in situ)	27176
Hemiepiphysiodesis (distal radius or ulna)	25450
Hemiepiphysiodesis (greater trochanter of the femur)	27185
Hemiepiphysiodesis (distal femur)	27475
Hemiepiphysiodesis (open, distal tibia)	27730
Tendon sheath excision (excision of the lesion of the tendon sheath, forearm, and/or wrist)	25110
Tendon sheath excision (excision of tendon sheath or joint capsule)	26160
Tendon sheath excision (excision of the lesion of the tendon sheath or capsule, leg, or/or ankle)	27630
Tendon sheath excision (excision of the lesion of the tendon sheath or capsule and foot)	28090
Fixation of femur shaft fracture (open treatment of femoral fracture)	27506
Fixation of femur shaft fracture (open treatment of femoral supracondylar or transcondylar fracture without intercondylar extension, including internal fixation)	27511
Fixation of femur shaft fracture (open treatment of femoral supracondylar or transcondylar fracture with intercondylar extension, including internal fixation)	27513
Excision of benign tumor of the femur (excision or curettage of bone cyst or benign tumor of the femur)	27355
Excision of benign tumor of the femur (excision or curettage of bone cyst or benign tumor of the tibia or fibula)	27635

We utilized the Physician Fee Schedule Look-Up Tool [8-10], from the Centers for Medicare and Medicaid Services (Baltimore County, MD), to input data reflecting the maximum fee that Medicare would pay physicians for the CPT codes between 2000 and 2022. We obtained prices from each CPT's Medicare Admissible Contractor Options and Modifier. The most recent consumer price index was adjusted for inflation and converted to 2022 US dollar (USD) by the US Department of Labor's Bureau of Labor Statistics [11].

Compound annual growth rates (CAGRs), a term used in investments to describe the rate of return over a period that dampens short-term periodic volatile changes, were calculated to measure the mean growth rate for each procedure and specialty [7]. Compound annual growth was then calculated by dividing the 2022 value by the value for 2000. We then raised the results to the power of 1 divided by the length of the time, and then, 1 was subtracted from the resulting value [12]. For each procedure, annual dollar changes were estimated using the previous years as a base. The decline in dollar value was found by subtracting the initial price of the surgical procedure in 2000 from the final year the price was recorded in 2022. The percent difference from its original price was found by taking that decline in dollar value price from the indicated years divided by the initial cost and then multiplied by 100 for the absolute percentage difference.

For each of the procedures of interest, a degree-5 polynomial regression model was fitted to the data to visualize the change in adjusted price over time.

## Results

**Figure 1:**
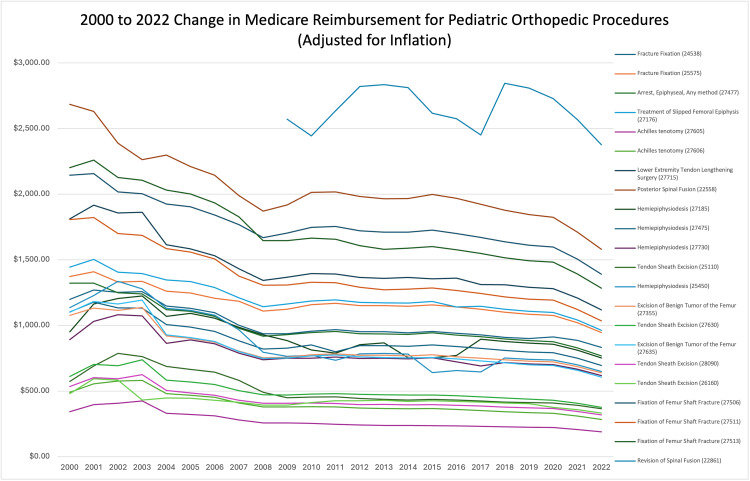
A year-by-year analysis of change in Medicare reimbursement for pediatric orthopedic procedures from 2000 to 2022 after adjusting for inflation Each line in the graph corresponds to one of the 22 pediatric orthopedic procedures evaluated in this study. The analysis spans the years 2000 to 2020, with one of the 22 procedures first being recorded in 2009 and continuing through 2022. The Y-axis reflects the dollar value adjusted for inflation, while the X-axis represents the yearly average dollar value over the specified period

Between 2000 and 2022, reimbursement decreased for all pediatric orthopedic procedures for their inflation-adjusted dollar amount and the CAGR. Between 2000 and 2022, all pediatric orthopedic procedures saw an overall decrease. These findings are demonstrated in Table 2 and in Figure 1, which demonstrates the year-by-year changes in Medicare reimbursement for the top procedures in pediatric orthopedics, adjusted for inflation from 2000 to 2022.

**Table 2:** The top pediatric orthopedic procedures and associated Current Procedural Terminology (CPT) codes, including the dollar value change, the CAGR, and total percent difference from the years 2000 to 2022 (adjusted for inflation for 2022 USD) *New code in 2009 CAGR, compound annual growth rate; USD, US dollar

CPT code	Procedure description	Average reimbursement in 2000	Average reimbursement in 2022	CAGR	Percent change from 2000 to 2022	Total price change from 2000 to 2022
22861	*Revision of spinal fusion	$1,885.80	$2,376.04	1.16%	-26.00%	$490.24
27176	Treatment of slipped femoral epiphysis	$849.36	$963.14	0.63%	-13.40%	$113.78
27185	Hemiepiphysiodesis	$951.83	$752.35	-1.17%	20.96%	-$199.48
24538	Fracture fixation	$1,198.27	$833.13	-1.80%	30.47%	-$365.14
27730	Hemiepiphysiodesis	$892.27	$618.31	-1.82%	30.70%	-$273.96
26160	Tendon sheath excision	$481.12	$332.74	-1.83%	30.84%	-$148.38
25575	Fracture fixation	$1,372.75	$925.37	-1.95%	32.59%	-$447.38
27506	Fixation of femur shaft fracture	$2,144.29	$1,390.13	-2.14%	35.17%	-$754.16
25110	Tendon sheath excision	$573.52	$366.34	-2.22%	36.12%	-$207.18
27475	Hemiepiphysiodesis	$1,102.83	$695.95	-2.28%	36.89%	-$406.88
27715	Lower extremity tendon lengthening surgery	$1,811.56	$1,118.03	-2.38%	38.28%	-$693.53
27630	Tendon sheath excision	$611.02	$375.76	-2.40%	38.50%	-$235.26
27635	Excision of benign tumor of the femur	$1,000.92	$606.64	-2.47%	39.39%	-$394.28
28090	Tendon sheath excision	$534.83	$319.64	-2.54%	40.24%	-$215.19
27355	Excision of benign tumor of the femur	$1,076.03	$639.06	-2.57%	40.61%	-$436.97
22558	Posterior spinal fusion	$2,685.71	$1,579.42	-2.62%	41.19%	-$1,106.29
27513	Fixation of femur shaft fracture	$2,201.98	$1,283.69	-2.66%	41.70%	-$918.29
27477	Arrest, epiphyseal, any method	$1,322.81	$768.20	-2.68%	41.93%	-$554.61
27606	Achilles tenotomy	$491.65	$284.46	-2.70%	42.14%	-$207.19
27511	Fixation of femur shaft fracture	$1,806.45	$1,035.66	-2.74%	42.67%	-$770.79
25450	Hemiepiphysiodesis	$1,136.14	$649.35	-2.76%	42.85%	-$486.79
27605	Achilles tenotomy	$417.27	$190.37	-3.85%	54.38%	-$226.90

Of all the procedures that we included in this study, Achilles tenotomy (local anesthesia CPT code 27605) saw the most significant overall decline in value at 54.37% from its original price, $229.90 less in reimbursement, and a 3.85% decrease in CAGR since the years 2000-2022.

The second procedure that saw a significant overall decline in value was hemiepiphysiodesis (distal radius or ulna CPT code 25450) with a decrease of 42.85% from its original price, $486.79 less in reimbursement, and a 2.76% decrease in CAGR since the years 2000-2020.

Of the CPT codes included in this analysis, only two featured a positive average inflation-adjusted reimbursement value. The first was the revision of spinal fusion (revision including the replacement of total disc arthroplasty CPT code 22861) at an increase of 25.99% from its original price, $490.24 more in reimbursement, and a 1.16% increase in CAGR since the years 2009-2022 (2009 was the first year this CPT code was introduced).

The second procedure that saw an increase was the treatment of slipped femoral epiphysis (by traction, without reduction, by single or multiple pinning, in situ, CPT code 27176) at 13.39% from its original price, $113.78 more in reimbursement, and a 0.63% CAGR since the years 2000-2022. The procedure with the most significant decline in dollar value from its original price was posterior spinal fusion (anterior approach for lumbar fusion CPT code 22558) with a decrease in reimbursement of $1106.29 since the years 2000-2022. Table 2 shows the average adjusted Medicare reimbursement for all 22 procedures.

Additionally, we completed an overall analysis of all procedures. The average reimbursement for all procedures decreased by 32.64%, with a loss of $383.85 in reimbursement and a decrease of 2.08% CAGR over the 22-year period examined in this study. This includes the procedure of the revision of spinal fusion (CPT code 22861), which began being utilized in 2009. The average reimbursement for all procedures without the "revision of spinal fusion (CPT code 22861)" saw a decrease of 35.43%, with a loss of $425.47 in reimbursement, and a decrease of 2.24% CAGR from the years 2000-2020. The inclusion of all 22 procedures is presented in Table 3 as the overall average percent change, the overall average total price change, and the overall average CAGR.

**Table 3:** The average of the overall percent change, overall total price change, and overall compound annual growth rate (CAGR) change from 2000 to 2022 (adjusted for inflation for 2022 USD) USD: US dollar

Overall average percent change from 2000 to 2020 for all procedures	Overall total price change from 2000 to 2022	Overall average CAGR change from 2000 to 2022
-32.65%	-$383.85	-2.08%

## Discussion

This study examined 10 of the most commonly performed procedures in pediatric orthopedic surgery and their variations for a total of 22 procedures. We recorded the billable codes for each surgery and identified the trends. We found that on average, Medicare reimbursement declined overall for 22 of the most common surgical procedures in pediatric orthopedic surgeries by 32.64% from 2000 to 2020, with adjustment for inflation.

Understanding procedural reimbursement trends is essential for maintaining medical practices' viability and for the growth of pediatric orthopedic surgical practices in the future. This study only looked at Medicare data's role in this analysis, which is publicly available. Medicare data had a lesser likelihood of local variation and showed greater consistency across areas. On a bigger scale, including commercial insurers would offer more data; however, the variance would be more challenging because of the state-to-state payment variation and payment rates for the contract surgeons within the same state, increasing difficulty in analysis.

At the time of data retrieval for this study, Medicare payments comprised an increasing percentage of pediatric orthopedic surgeons' overall reimbursement [13,14]. The trends reported within the analysis will consistently decline year over year in a significant portion of the CPT code surgeries. For the physician payment side, this can put them at a disadvantage in their practice and may have a concerning effect on their long-term viability into the future.

The implications of these findings are important due to the continuing growth of the pediatric population in the United States. The number of children in the United States continues to grow, reaching 74 million in 2021, which accounts for 22% of the nation's population [15]. The total number of children between the ages of zero and 17 has increased by a total of one million between 2000 and 2020 [16]. With the increase in the number of the pediatric population continuing to grow, the number of patients will follow, and the number of patients requiring pediatric orthopedic surgery will follow.

The decrease in reimbursement for pediatric orthopedic surgery discussed in this study can be explained in part by the congressional policy called the Balanced Budget Act of 1997 [17]. This cut took place under the sustainable growth rate legislation, which determines Medicare fee schedules [18]. This was proposed and passed to control Medicare spending on physician reimbursement to balance the federal budget during the year 2002 [17]. This played a role in the declining reimbursement across all physicians.

While this policy was placed over 20 years ago, it has had a lasting impact and demonstrates a clear effect on the trends of decreasing Medicare reimbursement in pediatric orthopedic surgery. This study looks at pediatric orthopedic surgery only; other surgical specialties also saw a similar decrease in reimbursement in their most commonly performed procedures, such as reconstructive plastic surgery, which saw a 14% decrease from 2000 to 2019 [19]; craniofacial trauma surgery from 2000 to 2021 saw a 16.6% decrease [20]. Also, literature regarding neurosurgery from 2000 to 2018 saw a decrease of 25.8% [21], and general surgery saw a 24.4% decrease from 2000 to 2018 [22]. These other studies provide further support of a declining trend in reimbursement structures in procedure-based care that we have also seen in this study on pediatric orthopedic surgery. This helps provide some explanation of the decrease in reimbursement demonstrated in our study and how Medicare is improperly evaluating the expertise of physicians.

While Medicare has devalued these procedures, many different factors may play a role in the decrease in reimbursement for procedures. Inpatient stays may be prolonged due to more invasive surgical interventions, while some patients may return home on the same day. Each of these variables may also play a role in the devaluation of reimbursement.

Creating different payment strategies to improve the financial alignment between physicians, hospitals, and payers will be vital for better reimbursing orthopedic physicians.

To address the constantly changing economic environment, better strategies leading to more stabilization and correcting this downward reimbursement trend should be attainable.

Private insurance companies often use Medicare valuation and reimbursement policies as benchmarks to charge the payers [23-25]. Our study looked at the trends in the top 10 procedures in pediatric orthopedic surgery and their variations based on CPT codes. Further analysis is required, as a prediction for the reimbursement of procedures should be continually revised. This analysis also did not address the changing political, economic, insurance, and technological influences that the future of orthopedic reimbursement may face. However, based on this study that incorporates the current Medicare data, it shows a consistent declining trend that can be predictive up until this point with the currently available data.

The future of reimbursement in pediatric orthopedic surgery and other specialties will require more quantitative information, research, and collaboration to find the most accurate way to evaluate reimbursement. The findings in this study bring forth the importance for those who make policies, those in charge of hospitals, and physicians as the starting point to develop proper valuation for reimbursement. The data presented in this study will serve as a guide to conduct proper evaluation and enable better direction of the appropriate policies aimed at ensuring fair reimbursement for pediatric orthopedic surgeons. This approach will be critical for addressing the observed decline in physician reimbursement and the implications within the broader healthcare system in the United States.

The limitations of this study include the focus on Medicare reimbursement data only. This is not a complete representation of the reimbursement of pediatric orthopedic surgeons, due to the use of private insurers. Another limitation of this study is that the data does not decide between each state, suggesting that location and region could play another role in reimbursement. This may also have an effect on different healthcare policies and the influence of local economic conditions that need to be considered. Additionally, this study examines the United States overall and does not offer specific data and trends that allow for local-scale analysis for regional- and state-focused trends. Further studies may be able to analyze more local-specific bias and provide more insight into the possible geographic variability to reimbursement trends.

## Conclusions

This investigation examined the trends in inflation-adjusted Medicare reimbursements from 2000 to 2022 for several of the most frequently performed procedures in pediatric orthopedic surgery, identified by their CPT codes. This study has shown that the largest mean decrease in reimbursement seen in pediatric orthopedic surgery procedures was with the Achilles tenotomy procedure. Raising awareness of these trends and understanding their implications are crucial for surgeons, policymakers, and hospital committees. Through further recognition, studies such as these could help set up future collaborative efforts in creating a comprehensive reimbursement model. Such a model could facilitate sustained growth and ensure ongoing access to high-quality pediatric orthopedic care across the United States.
